# Challenging trends in clinical-epidemiology and sero-prevalence of leptospirosis among febrile patients

**DOI:** 10.6026/973206300210736

**Published:** 2025-04-30

**Authors:** Akila Krishnamoorthy, Kamalraj Mohan, Sijimol Shanmughan, Subha Vajjiravel Jagganath, Anitha Srinivasagalu, Ambuja Sekhar, Senthamarai Srinivasan, Sivasankari Selvaraj

**Affiliations:** 1 Department of Microbiology, Meenakshi Medical College Hospital & Research Institute, Meenakshi Academy of Higher Education and Research (Deemed to be University), Kanchipuram-631552, Tamilnadu, India

**Keywords:** Leptospirosis, clinical manifestations, seroprevalence

## Abstract

Leptospirosis is the most prevalent zoonotic illness that affects both humans and animals worldwide. It is typically underreported due to a lack of knowledge, unusual manifestations and a lack of diagnostic resources. Therefore, it is of interest to estimate the seroprevalence of Leptospirosis from an acute febrile illness patient at a tertiary care hospital in India. Hence, a total of 252 blood samples were collected out of which 39 (15.47%) were positive for leptospirosis IgM antibody. Male patient (56.75%) shows preponderance over females. Further patients among the age group groups 21-40 (56.41%) showed the positivity. A clinical presentation such as Fever (100%), Myalgia (51.5%) and Headache (36.98%) was the most common symptoms seen followed by vomiting and abdominal pain. Leptospirosis screening must therefore be introduced as differential diagnosis in individuals with acute fever in addition to other routine testing in order to lower the morbidity and mortality.

## Background:

Leptospirosis is regarded as the most prevalent zoonotic illness worldwide, affecting both humans and animals, which accounts for around 10 lakh cases and 50,000 fatalities annually [1, 2]. The World Health Organization (WHO) says that the incidences vary from roughly 0.1 to 1 per 100,000 annually in temperate areas to 10-100 per 100,000 in humid climates [3].

Since 1931, cases of leptospirosis have been reported in India. Leptospirosis is rapidly becoming a major public health concern. Variable seroprevalence rates ranging from 6.4 to 30.9% have been reported by several investigations conducted across the nation [4]. There have been numerous reports of its occurrence in southern, central, eastern and western India in recent years because due to the intense monsoon, animal husbandry and unplanned urbanization [5]. Leptospira is spread by domestic and wild animals which comprises more than 14 infectious species with 250 serotypes [6]. Numerous studies have shown that the main risk factors for leptospirosis transmission, particularly in tropical nations, include residents of closeness to livestock ranches, paddy fields where one must walk and labor barefoot, sewers and floodwaters. Humans are unintentional hosts who become infected through direct or indirect contact with carrier animals' urine [7]. Additionally, in many regions of the nation, leptospirosis has long been identified as one of the main causes of acute febrile sickness [8]. The clinical condition will presenting with subclinical infections, Weil's syndrome and serious, life-threatening consequences. Classic symptoms, such as fever, myalgia, headache, and conjunctival irritation may extend to systemic complications includes Icterus, aseptic meningitis, suffusion, rash, hepatosplenomegaly, hemorrhagic signs, renal failure, pulmonary bleeding, and acute respiratory distress syndrome [9]. Leptospirosis has been underdiagnosed and underreported due to inadequate knowledge of the illness, epidemiological information and insufficiently skilled diagnostic facilities. Therefore, it is of interest to determine the clinical-epidemiological profile of leptospirosis cases reported as acute febrile illness (AFI) and to estimate the seroprevalence of among suspected AFI cases.

## Materials and Methods:

This study is a cross-sectional study carried out at Department of Microbiology, Meenakshi Medical College Hospital and Research Institute, Kanchipuram, India, over a period of one year from June'2022 to May'2023. Institutional ethical clearance (MMCH&RI IEC/Faculty/30/JUNE/22) was obtained from our institution and informed consent was taken from all the suspected patients with acute febrile illness. Patient attending with acute febrile illness with symptoms such as fever, headache, nausea and myalgia will be included in the study. Patient is not willing to participate in the study will be excluded.

## Sample collection:

A total of 3-5 ml of Blood samples will be collected aseptically from all the suspected cases of acute febrile illness. Serum will be separated and stored at -86°C for various serological diagnoses.

## ELISA for IgM antibodies to leptospira:

Extracted serum sample will be screened for the detection of specific IgM antibodies for Leptospirosis by using a commercial kit (Lepto IgM Microlisa, New Delhi, India) and interpreted according to the manufacturer's instructions.

## Processing and interpretation:

Add 125 µl Negative Control in A-1 and B-1 well. Add 125 µl Positive Control in C-1 well. Add 100 µl of sample diluent in each well, starting from D-1 followed by addition of 25µl of treated sample then cover and seal. Incubate at 37°C for 30 min. While the samples are incubating, prepare working Wash Solution and working Conjugate as specified in preparation of reagents. Take out the plate from the incubator after the incubation time is over and, wash the wells 5 times with working Wash Solution. Add 100 µl of working Conjugate Solution in each well. Apply cover & seal. Incubate at 37°C for 30 min. Aspirate and wash the wells 5 times with working Wash Solution. Add 100 µl of working substrate solution in each well. Incubate at room temperature (20-30°C) for 30 min. in dark. Add 50 µl of stop solution. Read absorbance at 450 nm and 630 nm within 30 minutes in ELISA reader.

## Statistical analysis:

IBM SPSS statistics software for Windows was used to do statistical analysis after data were loaded into Microsoft Excel sheets. Leptospirosis-positive cases were divided by suspected AFI cases to determine the seroprevalence between the various categories. The mean and Standard Deviation (SD) was used to display numerical data that was normally distributed. The level of significance was assessed using the Pearson Chi-square test and categorical data were displayed as percentages. A statistically significant p-value was defined.

## Results and Discussion:

A total of 252 blood samples collected from clinically suspected patients of acute febrile illness. Out of which 39(15.47%) were found to be positive for leptospirosis IgM Antibody. Majority of the acute febrile illness cases were seen in males 143(56.75%) than females were shown in Table 1. Among the age groups positivity were seen at the age between 21-40 (56.41%) years shown in Table 2. Rainfall exposure was found to be a significant risk factor and a significant portion of patients were identified as farmers (18%) followed by Fishermen's and labourer was illustrated in Table 3.

Clinical presentations such as Fever (100%), Myalgia (51.5%), Headache (36.98%) was the most common symptoms seen followed by vomiting, abdominal pain and conjunctival suffusion was shown in Table 4. This study also discovered seasonal fluctuation in leptospirosis infection. Monsoon period (46.18%) shows the maximum positivity rate followed by autumn (28.2%) shown in Table 5. However, it is due to waterlogging and contaminated water, which raises the risk of contact during this time. Co-morbidities also seen in few cases such as salmonellosis followed Dengue and Hepatitis B Viral Infection shown in Table 6. Leptospirosis is a bacterial zoonotic infection that has a high rate of morbidity and death. With the majority of cases coming from South America, the Caribbean and South Asia, it is one of the most common but underappreciated zoonosis [10]. Leptospirosis testing is not regular in clinical settings, unlike other infections that cause comparable symptoms, such as dengue, malaria and typhoid. The cause could be that clinicians are unaware of the disease's prevalence. There is only a few research reported from this region about the prevalence of leptospirosis [11]. In our study the prevalence rate is 15.4% which indicates the increasing trends of Leptospirosis in Tamilnadu at the year of 2022: a seroprevalence of seventeen per cent was seen in the study done by Kumari *et al.* 2015 [12]. However, kaur *et al.* reported high prevalence rate (17.2 %) in a study from Punjab. The study found that males accounted for almost two-thirds of leptospirosis cases. Males had a significantly higher seroprevalence rate (18.1%) than females (11.9%), with a p-value of 0.037. kaur *et al.* also reported as 20% of the male predominance than the female (18%) [13]. However, few study reported that male constitutes for 60% than females [14].

Males are more likely to be exposed to risks owing to outdoor activities and occupations. In this study, leptospirosis positive cases ranged from 21 to 40 years old, with a mean age of 32.5±13.3 years. Thus the 20-40 age groups have significantly greater seroprevalence compared to other groups. Kumari *et al.* found that patients with leptospirosis had an average age of 36.4 years, which is consistent with current findings [12]. However, Regmi *et al.* also shows the similar findings with the age group 30 to 40 [19]. Leptospirosis cases were primarily reported in agriculture and poultry. Our study shows 56.4% of those infected with leptospirosis were farmers followed by poultry and fisheries, whereas few study shows 33.9% in fishing is the most common occupation followed by agriculture (19.3%) [15]. Leptospirosis' peculiar presenting signs and clinical aspects make it similar to many other diseases. In the current study, fever (100%) was the most common symptom reported in all cases, followed by myalgia (51%), headache (36%), nausea/vomiting (31%) and abdominal pain (18%). Sethi *et al.* found a similar pattern in their study, however fever, myalgia and oliguria were the most common signs and symptoms recorded [17]. Seasonal fluctuation was seen in this study. Leptospirosis seroprevalence was shown to be greater during the monsoon season, consistent with prior investigations [16]. The high prevalence of infection during these months suggests that rain and moisture facilitate the spread of illness and Leptospira survival in soil. Study participants were asked about their exposure to a variety of risk factors. In our study, comorbidities were seen in few cases with salmonellosis (5%) followed by Dengue viral infection (5.1%) and Hepatitis B viral infection (2.5%). Gancheva *et al.* shows the similarity with several comorbidities such as viral hepatitis followed by pneumonia and cystitis [18]. However, laboratory studies of clinically suspected cases are necessary to confirm the diagnosis and initiate early therapy.

## Limitations of the study:

A significant number of leptospirosis infections have modest symptoms and are subclinical. Healthcare facilities typically do not record these incidents. The current study would therefore understate the prevalence of leptospirosis in the population.

IgM ELISA is a genus-specific test that is unable to identify a particular Leptospira serotype. However, especially in environments with limited resources, serological assays like ELISA are practical and efficient for measuring seroprevalence.

## Conclusion:

Leptospirosis is becoming the leading cause of acute febrile fever in several parts of India. The clinical diagnosis of leptospirosis and other acute febrile illnesses is difficult due to their nonspecific and overlapping clinical characteristics. Thus, in addition to other standard testing, leptospirosis screening must be added as a differential diagnosis in patients with acute fever in order to reduce the morbidity and mortality.

## Figures and Tables

**Table 1:** Gender wise distribution of the acute febrile illness patients

**Gender**	**Number**	**Positivity and Percentage**
Male	143	26 (18.1%)
Female	109	13 (11.9%)
Total	252	39 (100%)

**Table 2:** Age distribution of study population

**Age in years**	**Participants Number (%)**	**Positive Number (%)**
20-Jan	12(4.76)	1(2.56%)
21-40	133(52.78)	22 (56.41%)
41-60	82(32.54)	13(33.34%)
61-80	25(9.92)	3(7.69%)

**Table 3:** Occupation of the acute febrile illness patients

**Occupation**	**No. of patients**	**No. of Positive (%)**
Farmer	89	22(56.4%)
Labour	67	09 (23.07%)
Fishing	41	05 (13%)
Sweeper /Garbage cleaner	33	02(5.1%)
Others	22	01(2.6%)

**Table 4:** Clinical features of the acute febrile illness patients

**Signs and Symptoms**	**No. of patients**	**Percentage %**
Fever	252	100
Myalgia	131	51.5
Headache	92	36.98
Vomiting/diarrhoea	79	31.34
Abdominal pain	47	18.65
Jaundice	19	7.53
Hepatomegaly	86	34.12
Oliguria	17	6.74
Conjunctival suffusion	76	30.15

**Table 5:** Seasonal variation seen in patients with leptospirosis

**Seasonal variation**	**No. of positive (%)**
Summer	3(7.68)
Monsoon	18(46.18)
Autumn	11(28.20)
Winter	7(17.94)

**Table 6:** Co-morbidities present in leptospirosis Patients

**Co-morbidities**	**No. of positive**
Salmonellosis	02(5.1%)
Dengue fever	02(5.1%)
Hepatitis B	01(2.6%)
